# Methylated Organic Metabolites of Arsenic and their Cardiovascular Toxicities

**DOI:** 10.5487/TR.2008.24.3.161

**Published:** 2008-09-01

**Authors:** Ok-Nam Bae, Kyung-Min Lim, Ji-Yoon Noh, Keun-Young Kim, Eun-Kyung Lim, Jin-Ho Chung

**Affiliations:** College of Pharmacy, Institute of Pharmaceutical Sciences, Seoul National University, Seoul, 151-742 Korea

**Keywords:** Arsenic, Trivalent arsenic metabolite, MMA^III^, DMA^III^, Toxicity

## Abstract

Recently, arsenic-toxicity has become the major focus of strenuous assessment and dynamic research from the academy and regulatory agency. To elucidate the cause and the mechanism underlying the serious adverse health effects from chronic ingestion of arsenic-contaminated drinking water, numerous studies have been directed on the investigation of arsenic-toxicity using various *in vitro* as well as *in vivo* systems. Neverthless, some questions for arsenic effects remain unexplained, reflecting the contribution of unknown factors to the manifestation of arsenic-toxicity. Interestingly, very recent studies on arsenic metabolites have discovered that trivalent methylated arsenicals show stronger cytotoxic and genotoxic potentials than inorganic arsenic or pentavalent metabolites, arguing that these metabolites could play a key role in arsenic-associated disorders. In this review, recent progress and literatures are summarized on the metabolism of trivalent methylated metabolites and their toxicity on body systems including cardiovascular system in an effort to provide an insight into the future research on arsenic-associated disorders.

**Chemical properties of arsenic.** Arsenic is a naturally occurring metalloid, ubiquitously distributed in earth crust. (Smedley and Kinniburgh, 2002). The chemical properties of arsenic are complicated and a variety of chemical forms can be produced. Inorganic arsenic is formed through the reactions with oxygen, chloride and sulfur while organic arsenic is produced by combination with hydrogen or carbon (Oremland and Stolz, 2003). Arsenic can display 4 oxidative states, -3, 0, +3 and +5. Generally, trivalent arsenic is known to exhibit more toxicities than pentavalent arsenic species (Lee *et al.*, 1988). In natural state, arsenic exists in compound forms with copper, nickel and iron but in drinking water, arsenic is present mostly in the forms of inorganic pentavalent arsenate ($As^{V}$) or trivalent arsenite ($As^{III}$). Methylated organic arsenic is also found naturally and it is from the metabolism of microorganism (Oremland and Stolz, 2003).

**Arsenic exposure to human by drinking water.** Generally, the main source of arsenic exposure to human is contaminated drinking water and food (Gebel, 1999). Exposure via the contaminated food is mainly from arsenobetaine, arsenocholine and arsenosugars in sea foods (Edmonds *et al.*, 1997). However, due to their low toxicities and rapid urinary excretion, their effects on human are known to be minor (Buchet *et al.*, 1994). On the contrary, chronic arsenic exposure via contaminated drinking water is drawing an enormous public attention worldwide. (Abernathy *et al.*, 2003; Mead, 2005). US Environmental Protection Agency (EPA) and World Health Organization (WHO) are regulating the arsenic level in drinking water up to 10 μg/l (ppb) in an effort to prevent chronic arsenic exposure but the reports of arsenic exposure through contaminated drinking water are continuing throughout world. In a certain region of China, 0.09~1.86 mg/l level of arsenic was detected in the drinking water from a natural well (Xia and Liu, 2004) and in Bengal Delta Plain surrounded by India, West Bengal and Bangladesh, extremely high levels of arsenic ranging up to 3.2 mg/l have been reported (Bhattacharyya *et al.*, 2003). 3.05 mg/l arsenic level was detected in the underground water from a region of Vietnam (Berg *et al.*, 2001), and 0.01~1.82 mg/l of arsenic level was reported in some regions of Taiwan (Bates *et al.*, 1992). These epidemic arsenic contaminations have been also reported in other parts of world such as Latin America including Chile, Argentina and Mexico (Cebrian *et al.*, 1983; Hopenhayn-Rich *et al.*, 1996; Caceres *et al.*, 2005,), America and Europe (Lewis *et al.*, 1999; Lamm *et al.*, 2004).

**Distribution, metabolism and excretion of arsenic.** Arsenic in drinking water is mainly inorganic form and it can be easily absorbed through gastrointestinal tract to systemic blood circulation. Orally administered arsenic is found to be rapidly absorbed and cleared from body in human volunteer through urine for 3~4 days. However, some remain in body and are slowly excreted through hair, nail and sweat (Buchet *et al.*, 1981).

Arsenic distribution in blood has been investigated through the bioanalysis of arsenic concentration in plasma, serum and erythrocytes of arsenic exposed human (Heydorn, 1970; Concha *et al.*, 1998). Normal healthy people without arsenic exposure have the blood arsenic concentration less than 5.1 μg/l while arsenic-exposed group showed arsenic concentration of around 32.7 μg/l in erythrocytes and 15.4 μg/l in plasma (Heydorn, 1970). In addition, female population exposed to approximately 200 μg/l of arsenic contaminated drinking water showed average blood arsenic level of 11 μg/l (Concha *et al.*, 1998). Even after 2 years of wash-out period from arsenic exposure, plasma and blood concentration of arsenic were determined to be 7.48 μg/l and 26.3 μg/l, respectively (Mandal *et al.*, 2004). Other study in the population exposed to 5~410 μg/l of arsenic contaminated drinking water showed blood arsenic concentration of 2~42.1 μg/l (Olguin *et al.*, 1983; Pi *et al.*, 2000).

Other than blood, arsenic is mainly distributed into liver, hair and nails. Arsenic is mainly metabolized in liver and plasma undergoing reduction from +5 to +3 and subsequent oxidative methylation (Aposhian, 1997; Vahter, 2002; Aposhian *et al.*, 2004). Pentavalent oxidative state, arsenate ($As^V$) is transported into cell via phosphate carrier system and trivalent oxidative state, arsenite ($As^{III}$) is delivered via aquaglyceroporin (Liu *et al.*, 2002). Recently new metabolic pathways of inorganic arsenic have been suggested by Hayakawa *et al.* (2005). In cell, $As^V$ is reduced into $As^{III}$ in the presence of endogenous inosine or free thiol by purine nucleoside phosphorylase (PNP), glutathione-S-transferase (GST-omega) and unknown mitochondria enzymes (Radabaugh and Aposhian, 2000; Gregus and Nemeti, 2002; Nemeti and Gregus, 2002). Reduced $As^{III}$ forms a complex with glutathione (GSH) producing arsenic triglutathione (ATG), and ATG is methylated to monomethylarsenic diglutathione (MADG) and dimethylarsinic glutathione (DMAG) by arsenic methyltransferase (Cyt 19) using cofactor, S-adenosylmethione (SAM). MADG and DMAG are converted to $MMA^{III}$ and $DMA^{III}$ depending on the GSH concentration, and $MMA^{III}$ and $DMA^{III}$ are oxidized to $MMA^V$ and $DMA^V$ (Fig. 1).

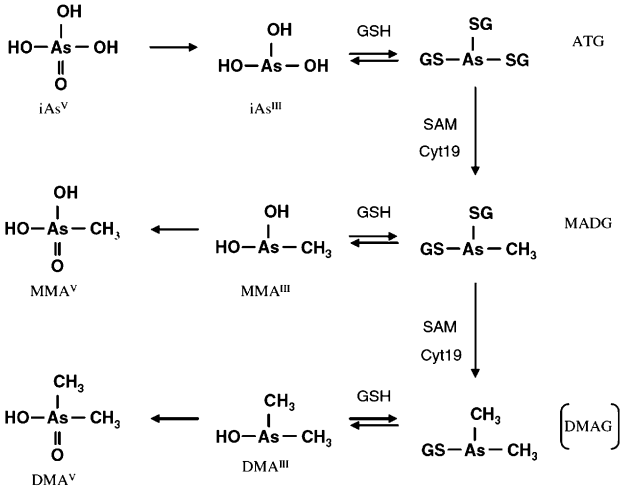

**Fig. 1.** Biotransformation of inorganic arsenic. $DMA^{III}$, dimethylarsinous acid; $DMA^V$, dimethylarsinic acid; $MMA^{III}$, monomethylarsonous acid; $MMA^V$, monomethylarsonic acid. ATG, arsenic triglutathione; MADG, monomethylarsenic diglutathione; DMAG, dimethylarsinic glutathione. Adapted from Hayakawa *et al.* (2005).

Major arsenic metabolite in urine is known to be $DMA^V$ but trivalent state was recently identified to be another major metabolite of arsenic in urine (Aposhian *et al.*, 2000a; Aposhian *et al.*, 2000b; Mandal *et al.*, 2004). In human urine, arsenic metabolite contents were measured to be in the order of $DMA^{III} > DMA^V > As^{III} > As^V > MMA^{III} > MMA^V$ (Valenzuela *et al.*, 2005). Although the actual level of trivalent methylated metabolites in environment and human tissue is still poorly investigated, several reports have dealt with the exposure level using *in vitro* and *in vivo* models. Upto now, $MMA^{III}$ and $DMA^{III}$ are known to be present in contaminated water (Hasegawa, 1994), hamster liver tissue (Sampayo-Reyes *et al.*, 2000), cultured human hepatocyte (Del Razo *et al.*, 2001), and human urine (Le *et al.*, 2000a, b; Del Razo *et al.*, 2001). In addition, the presence of $MMA^{III}$ in bile acid were reported in rats (Suzuki *et al.*, 2001). Tissue distribution of radioactive $DMA^{III}$ was also examined in mouse model (Hughes *et al.*, 2008).

**Toxicity of trivalent arsenic metabolites.** Biological activities of $MMA^V$ and $DMA^V$ are known to be inferior to those of inorganic arsenic and therefore, methylation pathways have been regarded as the detoxification of inorganic arsenic (Vahter and Marafante, 1983; Eguchi *et al.*, 1997; Del Razo *et al.*, 2001). Although trivalent methylated metabolites, $MMA^{III}$ and $DMA^{III}$ had been known to be chemically reactive (Cullen *et al.*, 1984), their toxicological roles were questioned due to the absence of knowledge about their biological concentrations. With the recent discovery of trivalent methylated metabolites, $MMA^{III}$ and $DMA^{III}$ as major human metabolites of inorganic arsenic and their strong toxicities, however, a renewed view of arsenic methylated metabolites as toxic metabolites is gaining more and more strength (Petrick *et al.*, 2000, 2001; Styblo *et al.*, 2000; Sakurai *et al.*, 2002). Petrick *et al.* (2000) showed that in Chang human hepatocyte, $MMA^{III}$ showed 10 times stronger toxicities than $As^{III}$ and potent toxicites of $MMA^{III}$ and $DMA^{III}$ could be also observed in other cell types such as normal human hepatocyte, epidermal keratinocyte, bronchial epithelial cell and urinary bladder UROtsa cell (Styblo *et al.*, 2000). In addition, toxicities of $MMA^{III}$ and $DMA^{III}$ were shown in various cancer cell lines including human hepatocellular carcinoma, human bladder transient carcinoma, human acute promyelocytic leukemia and human osteocarcinoma (Styblo *et al.*, 2002) and inhibition potency of $MMA^{III}$ against glutathione reductase, glutathione peroxidase and thioredoxin reductase was also the strongest among arsenic species (Chouchane and Snow, 2001; Lin *et al.*, 2001). $DMA^{III}$ is known to have neurotoxic potential in contrast to $DMA^{V}$ (Kruger *et al.*, 2007). Furthermore, trivalent methylated arsenic species are known to directly react with DNA to induce genotoxicity (Mass *et al.*, 2001) and they have been identified as clastogen in human lymphocyte and mouse lymphoma cell (Kligerman *et al.*, 2003). Indeed, after intraperitoneal injection, $MMA^{III}$ showed strong acute toxicities in hamsters. $LD_{50}$ level of $MMA^{III}$ was estimated to be around 29.3 mmol/kg which is 5 times lower than that of inorganic arsenic (Petrick *et al.*, 2001).

The reason for the potent toxicities of trivalent methylated metabolites over inorganic or pentavalent methylated metabolites has not been fully elaborated, but several explanations such the higher affinity to free thiol (Shiobara *et al.*, 2001) and the strong potential of oxidative stress (Nesnow *et al.*, 2002) of trivalent methylated metabolite have been suggested. Some reports on the thiol-bound arsenicals suggested that thiol-bound form, which is referred as arsenothiol, could be responsible for the toxicity of methylated metabolites (Styblo *et al.*, 1997). Supporting this view, a recent paper has shown that low-concentration of dithiol compounds potentiate the toxicity of trivalent methylated metabolites (Jan *et al.*, 2006). In addition, higher intracellular accumulation of $MMA^{III}$ over $As^{III}$ might be suggested as well for the strong toxicity of $MMA^{III}$ (Hirano *et al.*, 2004).

**Effects of trivalent arsenic metabolites on cardiovascular system.** Based on the epidemiological reports on the link between chronic ingestion of arsenic-contaminated drinking water and diverse cardiovasular diseases inculding peripheral vascular disease, ischemic heart disease, atherosclerosis and hypertension (Yu *et al.*, 2002; Abernathy *et al.*, 2003; Tseng *et al.*, 2003; Simeonova and Luster, 2004; Yoshida *et al.*, 2004; Kwok *et al.*, 2007), numerous studies have been directed to elucidate the effects of arsenic on cardiovascular system using *in vitro* as well as *in vivo* assays. It is now well-established by substantial number of evidences that the inorganic arsenic including arsenite and arsenate has deleterious effects on cardiovascular system. Exposure to arsenite and arsenate could lead to the development of atherosclerosis, increase of reactive nitrogen species, thrombus formation, and decrease of nitric oxide and cyclic GMP *in vivo* (Lee *et al.*, 2002; Simeonova *et al.*, 2003; Bunderson *et al.*, 2004). In isolated vascular tissues, alteration of vascular tone was induced by arsenite-induced excessive contraction as well as impaired relaxation (Lee *et al.*, 2003, 2005). In vascular cells like vascular smooth muscle cells and endothelial cells, inorganic arsenic could induce oxidative stress, DNA damage and apoptosis with the expression of several proteins by activation of transcription factors (Hirano *et al.*, 2003; Tsou *et al.*, 2003; Kumagai and Pi, 2004; Soucy *et al.*, 2004). Inorganic arsenic also enhances the activation of platelets, potentiating agonist-induced platelet aggregation and procogulant activity through phosphatidylserine exposure and microparticle generation (Lee *et al.*, 2002; Bae *et al.*, 2007).

Although a wide range of cardiovascular effects of inorganic arsenic is well-characterized, information on the role of trivalent methylated metabolites in arsenic-associated diseases is extremely limited. Hirano *et al.* (2004) showed that $MMA^{III}$ has higher toxicity than other arsenic species in rat heart endothelial cells, and Li *et al.* (2007) have reported that $MMA^{III}$ exposure leads to the inhibition of eNOS activity and alteration of eNOS phosphorylation in endothelial cells. Recently, we investigated the effects of trivalent methylated metabolites on vascular tone using isolated blood vessel, and found that $MMA^{III}$ and $DMA^{III}$ could induce smooth muscle dysfunction through the impairment of agonist-induced blood vessel contraction, which is mediated by the disturbance of calcium regulation (Fig. 2). Dysfunction of smooth muscle cells is well-correlated with the alteration of blood pressure after exposure to $MMA^{III}$ in *in*

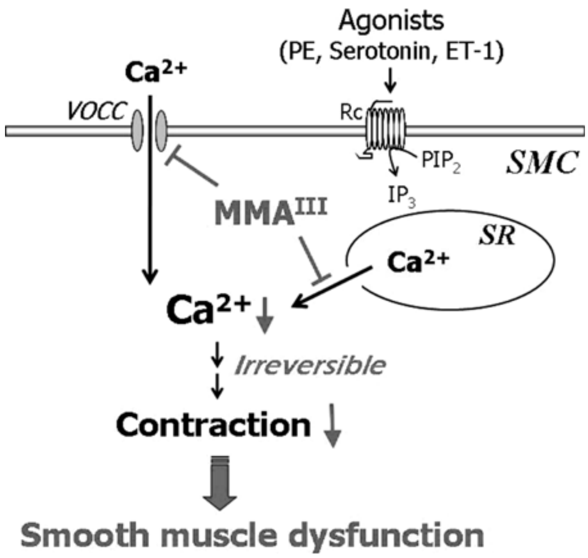

**Fig. 2.** A suggested mechanism for smooth muscle dysfunction by $MMA^{III}$. SMC, smooth muscle cells; SR, sarcoplasmic reticulum; VOCC, voltage-operated calcium channel. Adapted from Bae *et al*. (2008).

*vivo* animal model. Besides vascular cells like smooth muscle cells or endothelial cells, trivalent methylated arsenic metabolites could affect platelet function. In our recent study, $MMA^{III}$ and $DMA^{III}$ were shown to induce apoptotic-like events in platelets, accompanied by phosphatidylserine exposure, resulting in the promotion of procoagulant activity which could be also confirmed in *in vivo* thrombosis model (unpublished data). Notably, procoagulant activity could be increased directly by $MMA^{III}$ and $DMA^{III}$ in the absence of activating agonists, in contrast to the case of inorganic arsenic where agonist was required for the inorganic arsenic-induced procoagulant activity (Bae *et al*., 2007).

**Concluding remarks.** Due to the potent toxicity and/or high reactivity of trivalent methylated arsenics *in vitro*, increasing efforts are currently being made to explain the role of these metabolites for the arsenic-associated adverse health effects. Although their actual level in environment and body after the chronic ingestion of arsenic need to be addressed further to confirm their roles, it is a matter of fact that trivalent methylated arsenic metabolites have deleterious effects on the diverse body systems, especially cardiovascular system. Upto now, most of the toxicological studies for the regulatory purposes have been focused on the effects of inorganic arsenic, but now the bottom line for assessment of arsenic-toxicity should be changed to explore the possible involvement of trivalent methylated metabolites. The potent effects of trivalent methylated metabolites with distinct toxic mechanisms from that of inorganic arsenic could give a novel clue for the explanation of the unsolved questions for arsenic-associated health effects.

## ACKNOWLEDGEMENTS

This work was supported by the Korea Research Foundation Grant funded by the Korean Government (MOEHRD) (KRF-2006-E00162).

## References

[CR1] Abernathy CO, Thomas DJ, Calderon RL (2003). Health effects and risk assessment of arsenic. J. Nutr..

[CR2] Aposhian HV (1997). Enzymatic methylation of arsenic species and other new approaches to arsenic toxicity. Annu. Rev. Pharmacol. Toxicol..

[CR3] Aposhian HV, Gurzau ES, Le XC, Gurzau A, Healy SM, Lu X, Ma M, Yip L, Zakharyan RA, Maiorino RM, Dart RC, Tircus MG, Gonzalez-Ramirez D, Morgan DL, Avram D, Aposhian MM (2000). Occurrence of monomethylarsonous acid in urine of humans exposed to inorganic arsenic. Chem. Res. Toxicol..

[CR4] Aposhian HV, Zheng B, Aposhian MM, Le XC, Cebrian ME, Cullen W, Zakharyan RA, Ma M, Dart RC, Cheng Z, Andrewes P Y L, O’Malley GF, Maiorino RM, Van Voorhies W, Healy SM, Titcomb A (2000). DMPS-arsenic challenge test. II. Modulation of arsenic species, including monomethylarsonous acid (MMA(III)), excreted in human urine. Toxicol. Appl. Pharmacol..

[CR5] Aposhian HV, Zakharyan RA, Avram MD, Sampayo-Reyes A, Wollenberg ML (2004). A review of the enzymology of arsenic metabolism and a new potential role of hydrogen peroxide in the detoxication of the trivalent arsenic species. Toxicol. Appl. Pharmacol..

[CR6] Bae ON, Lim EK, Lim KM, Noh JY, Chung SM, Lee MY, Yun YP, Kwon SC, Lee JH, Nah SY, Chung JH (2008). Vascular smooth muscle dysfunction induced by monomethylarsonous acid (MMA^III^): A contributing factor to arsenic-associated cardiovascular diseases. Environ. Res..

[CR7] Bae ON, Lim KM, Noh JY, Chung SM, Kim H, Lee CR, Park JD, Chung JH (2007). Arsenite-enhanced procoagulant activity through phosphatidylserine exposure in platelets. Chem. Res. Toxicol..

[CR8] Bates MN, Smith AH, Hopenhayn-Rich C (1992). Arsenic ingestion and internal cancers: a review. Am. J. Epidemiol..

[CR9] Berg M, Tran HC, Nguyen TC, Pham HV, Schertenleib R, Giger W (2001). Arsenic contamination of groundwater and drinking water in Vietnam: a human health threat. Environ. Sci. Technol..

[CR10] Bhattacharyya R, Chatterjee D, Nath B, Jana J, Jacks G, Vanter M (2003). High arsenic groundwater: mobilization, metabolism and mitigation—an overview in the Bengal Delta Plain. Mol. Cell. Biochem..

[CR11] Buchet JP, Lauwerys R, Roels H (1981). Comparison of the urinary excretion of arsenic metabolites after a single oral dose of sodium arsenite, monomethylarsonate, or dimethylarsinate in man. Int. Arch. Occup. Environ. Health.

[CR12] Buchet JP, Pauwels J, Lauwerys R (1994). Assessment of exposure to inorganic arsenic following ingestion of marine organisms by volunteers. Environ. Res..

[CR13] Bunderson M, Brooks DM, Walker DL, Rosenfeld ME, Coffin JD, Beall HD (2004). Arsenic exposure exacerbates atherosclerotic plaque formation and increases nitrotyrosine and leukotriene biosynthesis. Toxicol. Appl. Pharmacol..

[CR14] Caceres DD, Pino P, Montesinos N, Atalah E, Amigo H, Loomis D (2005). Exposure to inorganic arsenic in drinking water and total urinary arsenic concentration in a Chilean population. Environ. Res..

[CR15] Cebrian ME, Albores A, Aguilar M, Blakely E (1983). Chronic arsenic poisoning in the north of Mexico. Hum. Toxicol..

[CR16] Chouchane S, Snow ET (2001). *In vitro* effect of arsenical compounds on glutathione-related enzymes. Chem. Res. Toxicol..

[CR17] Concha G, Vogler G, Lezcano D, Nermell B, Vahter M (1998). Exposure to inorganic arsenic metabolites during early human development. Toxicol. Sci..

[CR18] Cullen WR, McBride BC, Reglinski J (1984). The reaction of methylarsenicals with thiols: Some biological implications. J. Inorg. Biochem..

[CR19] Del Razo LM, Styblo M, Cullen WR, Thomas DJ (2001). Determination of trivalent methylated arsenicals in biological matrices. Toxicol. Appl. Pharmacol..

[CR20] Edmonds JS, Shibata Y, Francesconi KA, Rippingale RJ, Moritaet M (1997). Arsenic transformations in short marine food chains studied by HPLC-ICP MS. Appl. Organomet. Chem..

[CR21] Eguchi N, Kuroda K, Endo G (1997). Metabolites of arsenic induced tetraploids and mitotic arrest in cultured cells. Arch. Environ. Contam. Toxicol..

[CR22] Gebel TW (1999). Arsenic and drinking water contamination. Science.

[CR23] Gregus Z, Nemeti B (2002). Purine nucleoside Phosphorylase as a cytosolic arsenate reductase. Toxicol. Sci..

[CR24] Hasegawa H, Sohrin Y, Matsui M, Hojo M, Kawashima M (1994). Speciation of arsenic in natural waters by solvent extraction and hydride generation atomic absorption spectrometry. Anal. Chem..

[CR25] Hayakawa T, Kobayashi X, Cui X, Hirano S (2005). A new metabolic pathway of arsenite: arsenic-glutathione complexes are substrates for human arsenic methyltransferase Cyt19. Arch. Toxicol..

[CR26] Heydorn K (1970). Environmental variation of arsenic levels in human blood determines by neutron activation analysis. Clin. Chim. Acta.

[CR27] Hirano S, Cui X, Li S, Kanno S, Kobayashi Y, Hayakawa T, Shraim A (2003). Difference in uptake and toxicity of trivalent and pentavalent inorganic arsenic in rat heart microvessel endothelial cells. Arch. Toxicol..

[CR28] Hirano S, Kobayashi Y, Cui X, Kanno S, Hayakawa T, Shraim A (2004). The accumulation and toxicity of methylated arsenicals in endothelial cells: important roles of thiol compounds. Toxicol. Appl. Pharmacol..

[CR29] Hopenhayn-Rich C, Biggs ML, Fuchs A, Bergoglio R, Tello EE, Nicolli H, Smith AH (1996). Bladder cancer mortality associated with arsenic in drinking water in Argentina. Epidemiology.

[CR30] Hughes MF, Devesa V, Adair BM, Conklin SD, Creed JT, Styblo M, Kenyon EM, Thomas DJ (2008). Tissue dosimetry, metabolism and excretion of pentavalent and trivalent dimethylated arsenic in mice after oral administration. Toxicol. Appl. Pharmacol..

[CR31] Jan KY, Wang TC, Ramanathan B, Gurr JR (2006). Dithiol compounds at low concentrations increase arsenite toxicity. Toxicol. Sci..

[CR32] Kligerman AD, Doerr CL, Tennant AH, Harrington-Brock K, Allen JW, Winkfield E, Poorman-Allen P, Kundu B, Funasaka K, Roop BC, Mass MJ, DeMarini DM (2003). Methylated trivalent arsenicals as candidate ultimate genotoxic forms of arsenic: induction of chromosomal mutations but not gene mutations. Environ. Mol. Mutagen..

[CR33] Kruger K, Repges H, Hippler J, Hartmann LM, Hirner AV, Straub H, Binding N, Musshoff U (2007). Effects of dimethylarsinic and dimethylarsinous acid on evoked synaptic potentials in hippocampal slices of young and adult rats. Toxicol. Appl. Pharmacol..

[CR34] Kumagai Y, Pi J (2004). Molecular basis for arsenic-induced alteration in nitric oxide production and oxidative stress: implication of endothelial dysfunction. Toxicol. Appl. Pharmacol..

[CR35] Kwok RK, Mendola P, Liu ZY, Savitz DA H G, Ling HL, Xia Y, Lobdell D, Zeng D, Thorp JM, Creason JP, Mumford JL (2007). Drinking water arsenic exposure and blood pressure in healthy women of reproductive age in Inner Mongolia, China. Toxicol. Appl. Pharmacol..

[CR36] Lamm SH, Engel A, Kruse MB, Feinleib M, Byrd DM, Lai S, Wilson R (2004). Arsenic in drinking water and bladder cancer mortality in the United States: an analysis based on 133 U.S. counties and 30 years of observation. J. Occup. Environ. Med..

[CR37] Le XC, Lu X, Ma M, Cullen WR, Aposhian HV, Zheng B (2000). Speciation of key arsenic metabolic intermediates in human urine. Anal. Chem..

[CR38] Le XC, Ma M, Cullen WR, Aposhian HV, Lu X, Zheng B (2000). Determination of monomethylarsonous acid, a key arsenic methylation intermediate, in human urine. Environ. Health Perspect..

[CR39] Lee TC, Tanaka N, Lamb PW, Gilmer TM, Barrett JC (1988). Induction of gene amplification by arsenic. Science.

[CR40] Lee MY, Bae ON, Chung SM, Kang KT, Lee JY, Chung JH (2002). Enhancement of platelet aggregation and thrombus formation by arsenic in drinking water: a contributing factor to cardiovascular disease. Toxicol. Appl. Pharmacol..

[CR41] Lee MY, Jung BI, Chung SM, Bae ON, Lee JY, Park JD, Yang JS, Lee H, Chung JH (2003). Arsenic-induced dysfunction in relaxation of blood vessels. Environ. Health Perspect..

[CR42] Lee MY, Lee YH, Lim KM, Chung SM, Bae ON, Kim H L CR, Park JD, Chung JH (2005). Inorganic arsenite potentiates vasoconstriction through calcium sensitization in vascular smooth muscle. Environ. Health Perspect..

[CR43] Lewis DR, Southwick JW, Ouellet-Hellstrom R, Rench J, Calderon RL (1999). Drinking water arsenic in Utah: A cohort mortality study. Environ. Health Perspect..

[CR44] Li B, Sun Y, Sun X W Y, Li X, Kumagai Y, Sun G (2007). Monomethylarsonous acid induced cytotoxicity and endothelial nitric oxide synthase phosphorylation in endothelial cells. Bull. Environ. Contam. Toxicol..

[CR45] Lin S, Del Razo LM, Styblo M, Wang C, Cullen WR, Thomas DJ (2001). Arsenicals inhibit thioredoxin reductase in cultured rat hepatocytes. Chem. Res. Toxicol..

[CR46] Liu Z, Shen J, Carbrey JM, Mukhopadhyay R, Agre P, Rosen BP (2002). Arsenite transport by mammalian aquaglyceroporins AQP7 and AQP9. Proc. Natl. Acad. Sci. USA.

[CR47] Mandal BK, Ogra Y, Anzai K, Suzuki KT (2004). Speciation of arsenic in biological samples. Toxicol. Appl. Pharmacol..

[CR48] Mass MJ, Tennant A, Roop BC, Cullen WR, Styblo M, Thomas DJ, Kligerman AD (2001). Methylated trivalent arsenic species are genotoxic. Chem. Res. Toxicol..

[CR49] Mead, M.N. (2005). Arsenic: in search of an antidote to a global poison. *Environ. Health Perspect.*, **113**, A378-386. 10.1289/ehp.113-a378 PMC1257621 15929879

[CR50] Nemeti B, Gregus Z (2002). Mitochondria work as reactors in reducing arsenate to arsenite. Toxicol. Appl. Pharmacol..

[CR51] Nesnow S, Roop BC, Lambert G, Kadiiska M, Mason RP, Cullen WR, Mass MJ (2002). DNA damage induced by methylated trivalent arsenicals is mediated by reactive oxygen species. Chem. Res. Toxicol..

[CR52] Olguin A, Jauge P, Cebrian M, Albores A (1983). Arsenic levels in blood, urine, hair and nails from a chronically exposed human population. Proc. West Pharmacol. Soc.

[CR53] Oremland RS, Stolz JF (2003). The ecology of arsenic. Science.

[CR54] Petrick JS, Ayala-Fierro F, Cullen WR, Carter DE, Aposhian HV (2000). Monomethylarsonous acid (MMA(III)) is more toxic than arsenite in Chang human hepatocytes. Toxicol. Appl. Pharmacol..

[CR55] Petrick JS, Jagadish B, Mash EA, Aposhian HV (2001). Monomethylarsonous acid (MMA(III)) and arsenite: LD(50) in hamsters and in vitro inhibition of pyruvate dehydrogenase. Chem. Res. Toxicol..

[CR56] Pi J, Kumagai Y, Sun G, Yamauchi H, Yoshida T, Iso H, Endo A, Yu L, Yuki K, Miyauchi T, Shimojo N (2000). Decreased serum concentrations of nitric oxide metabolites among Chinese in an endemic area of chronic arsenic poisoning in inner Mongolia. Free Radic. Biol. Med..

[CR57] Radabaugh TR, Aposhian HV (2000). Enzymatic reduction of arsenic compounds in mammalian systems: reduction of arsenate to arsenite by human liver arsenate reductase. Chem. Res. Toxicol..

[CR58] Sakurai T, Qu W, Sakurai MH, Waalkes MP (2002). A major human arsenic metabolite, dimethylarsinic acid, requires reduced glutathione to induce apoptosis. Chem. Res. Toxicol..

[CR59] Sampayo-Reyes A, Zakharyan RA, Healy SM, Aposhian HV (2000). Monomethylarsonic acid reductase and monomethylarsonous acid in hamster tissue. Chem. Res. Toxicol..

[CR60] Shiobara Y, Ogra Y, Suzuki KT (2001). Animal species difference in the uptake of dimethylarsinous acid (DMA(III)) by red blood cells. Chem. Res. Toxicol..

[CR61] Simeonova PP, Hulderman T, Harki D, Luster MI (2003). Arsenic exposure accelerates atherogenesis in apolipoprotein E(-/-) mice. Environ. Health Perspect..

[CR62] Simeonova PP, Luster MI (2004). Arsenic and atherosclerosis. Toxicol. Appl. Pharmacol..

[CR63] Smedley PL, Kinniburgh DG (2002). A review of the source, behaviour and distribution of arsenic in natural waters. Appl. Geochem..

[CR64] Soucy NV, Klei LR, Mayka DD, Barchowsky A (2004). Signaling pathways for arsenic-stimulated vascular endothelial growth factor-a expression in primary vascular smooth muscle cells. Chem. Res. Toxicol..

[CR65] Styblo M, Del Razo LM, Vega L, Germolec DR, LeCluyse EL, Hamilton GA, Reed W, Wang C, Cullen WR, Thomas DJ (2000). Comparative toxicity of trivalent and pentavalent inorganic and methylated arsenicals in rat and human cells. Arch. Toxicol..

[CR66] Styblo M, Drobna Z, Jaspers I, Lin S, Thomas DJ (2002). The role of biomethylation in toxicity and carcinogenicity of arsenic: a research update. Environ. Health Perspect..

[CR67] Styblo M, Serves SV, Cullen WR, Thomas DJ (1997). Comparative inhibition of yeast glutathione reductase by arsenicals and arsenothiols. Chem. Res. Toxicol..

[CR68] Suzuki KT, Tomita T, Ogra Y, Ohmichi M (2001). Glutathione-conjugated arsenics in the potential hepato-enteric circulation in rats. Chem. Res. Toxicol..

[CR69] Tseng CH, Chong CK, Tseng CR, Hsueh YM, Chiou HY, Tseng CC, Chen CJ (2003). Long-term arsenic exposure and ischemic heart disease in arsenia-sis-hyperendemic villages in Taiwan. Toxicol. Lett..

[CR70] Tsou TC, Tsai FY, Wu MC, Chang LW (2003). The protective role of NF-kappaB and AP-1 in arsenite-induced apoptosis in aortic endothelial cells. Toxicol. Appl. Pharmacol..

[CR71] Vahter M (2002). Mechanisms of arsenic biotransformation. Toxicology.

[CR72] Vahter M, Marafante E (1983). Intracellular interaction and metabolic fate of arsenite and arsenate in mice and rabbits. Chem. Biol. Interact.

[CR73] Valenzuela OL, Borja-Aburto VH, Garcia-Vargas GG, Cruz-Gonzalez MB, Garcia-Montalvo EA, Calderon-Aranda ES, Del Razo LM (2005). Urinary trivalent methylated arsenic species in a population chronically exposed to inorganic arsenic. Environ. Health Perspect..

[CR74] Wildfang E, Radabaugh TR, Aposhian HV (2001). Enzymatic methylation of arsenic compounds. IX. Liver arsenite methyltransferase and arsenate reductase activities in primates. Toxicology.

[CR75] Xia Y, Liu J (2004). An overview on chronic arsenism via drinking water in PR China. Toxicology.

[CR76] Yoshida T, Yamauchi H, Fan Sun G (2004). Chronic health effects in people exposed to arsenic via the drinking water: dose-response relationships in review. Toxicol. Appl. Pharmacol..

[CR77] Yu HS, Lee CH, Chen GS (2002). Peripheral vascular diseases resulting from chronic arsenical poisoning. J. Dermatol..

[CR78] Zakharyan RA, Aposhian HV (1999). Arsenite methylation by methylvitamin B12 and glutathione does not require an enzyme. Toxicol. Appl. Pharmacol..

